# Anisotropic Nodal‐Line‐Derived Large Anomalous Hall Conductivity in ZrMnP and HfMnP

**DOI:** 10.1002/adma.202104126

**Published:** 2021-09-12

**Authors:** Sukriti Singh, Jonathan Noky, Shaileyee Bhattacharya, Praveen Vir, Yan Sun, Nitesh Kumar, Claudia Felser, Chandra Shekhar

**Affiliations:** ^1^ Max Planck Institute for Chemical Physics of Solids 01187 Dresden Germany; ^2^ Present address: Paul Scherrer Institut CH‐ 5232 Villigen‐PSI Switzerland; ^3^ Present address: Diffraction Group Institut Laue‐Langevin 71 Avenue des Martyrs Grenoble 38000 France; ^4^ Present address: S. N. Bose National Centre for Basic Sciences Salt Lake City Kolkata 700 106 India

**Keywords:** anomalous Hall effect, Berry curvatures, ferromagnets, mirror planes, nodal lines

## Abstract

The nontrivial band structure of semimetals has attracted substantial research attention in condensed matter physics and materials science in recent years owing to its intriguing physical properties. Within this class, a group of nontrivial materials known as nodal‐line semimetals is particularly important. Nodal‐line semimetals exhibit the potential effects of electronic correlation in nonmagnetic materials, whereas they enhance the contribution of the Berry curvature in magnetic materials, resulting in high anomalous Hall conductivity (AHC). In this study, two ferromagnetic compounds, namely ZrMnP and HfMnP, are selected, wherein the abundance of mirror planes in the crystal structure ensures gapped nodal lines at the Fermi energy. These nodal lines result in one of the largest AHC values of 2840 Ω^−1^ cm^−1^, with a high anomalous Hall angle of 13.6% in these compounds. First‐principles calculations provide a clear and detailed understanding of nodal line‐enhanced AHC. The finding suggests a guideline for searching large AHC compounds.

## Graphical abstract

Anomalous Hall conductivity appears in metallic ferromagnets due to the anomalous velocity of charge carriers in the presence of a magnetic field. In ferromagnets ZrMnP and HfMnP, the abundance of mirror planes forming nodal lines at the Fermi energy guides and enhances such velocity, resulting in both the compounds exhibiting large value of anomalous Hall conductivity.

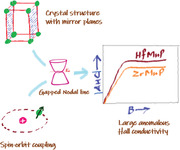

## 1 Introduction

Topological materials have attracted substantial attention owing to their nontrivial electronic band structures that offer the potential for revolutionary device applications. A nontrivial band topology appears when band inversion occurs in momentum space; that is, the conduction band is below the valence band with respect to their natural order. This inversion may occur in several manners, and the corresponding wave function of each band twists and induces a finite Berry phase that is associated with the Berry curvature (BC). In addition to the accidental touching of bands at a node, they may also form a line, and such compounds are known as nodal‐line compounds. The materials may be both magnetic and nonmagnetic, in which the nodal lines are protected by mirror symmetry,^[^ ^1^, ^2^, ^3^, ^4^, ^5^, ^6^, ^7^ ^]^ and consequently, a drum head‐like topological surface state exists.^[^ ^1^, ^2^, ^3^, ^4^ ^]^ In ferromagnets, all bands are usually singly degenerate but in the presence of mirror planes, they may be doubly degenerate in the form of a Dirac nodal line. In such materials, the magnetism and topology are entangled, and depending on the applied magnetic field direction, the degeneracy of the nodal line is lifted and a nontrivial gap is opened.^[^ ^8^, ^9^ ^]^ The BC sum over such gapped lines is enhanced significantly. The BC that is associated with nontrivial bands as a source of anomalous Hall conductivity (AHC) has recently been recognized in various compounds; for example, the chiral antiferromagnets Mn_3_Sn^[^ ^10^ ^]^ and Mn_3_Ge,^[^ ^11^ ^]^ kagome lattice ferromagnet Co_3_Sn_2_S_2_,^[^ ^12^ ^]^ nodal‐line ferromagnets Co_2_MnZ (Z = Ga, Al)^[^ ^8^, ^13^, ^14^ ^]^ and MnAlGe.^[^ ^15^ ^]^ The presence of nodal lines has been revealed experimentally by spectroscopy.^[^ ^15^, ^16^ ^]^ Among these, Co_2_MnZ has exhibited the record AHC value of 1600–2000 Ω^−1^ cm^−1^ at 2 K and many more compounds from the same family are awaiting experimental realization, which suggests the crucial role of mirror planes.^[^ ^9^ ^]^ Apart from the nontrivial band topology, an important factor for the occurrence of large AHC is spin–orbit coupling (SOC). We observed an AHC of 2000 Ω^−1^ cm^−1^ for ZrMnP and 2840 Ω^−1^ cm^–1^ for HfMnP. In this work, our approach is to study the AHC in systems with large SOC and that contain ample mirror planes in the crystal structures.

**Figure 1:**
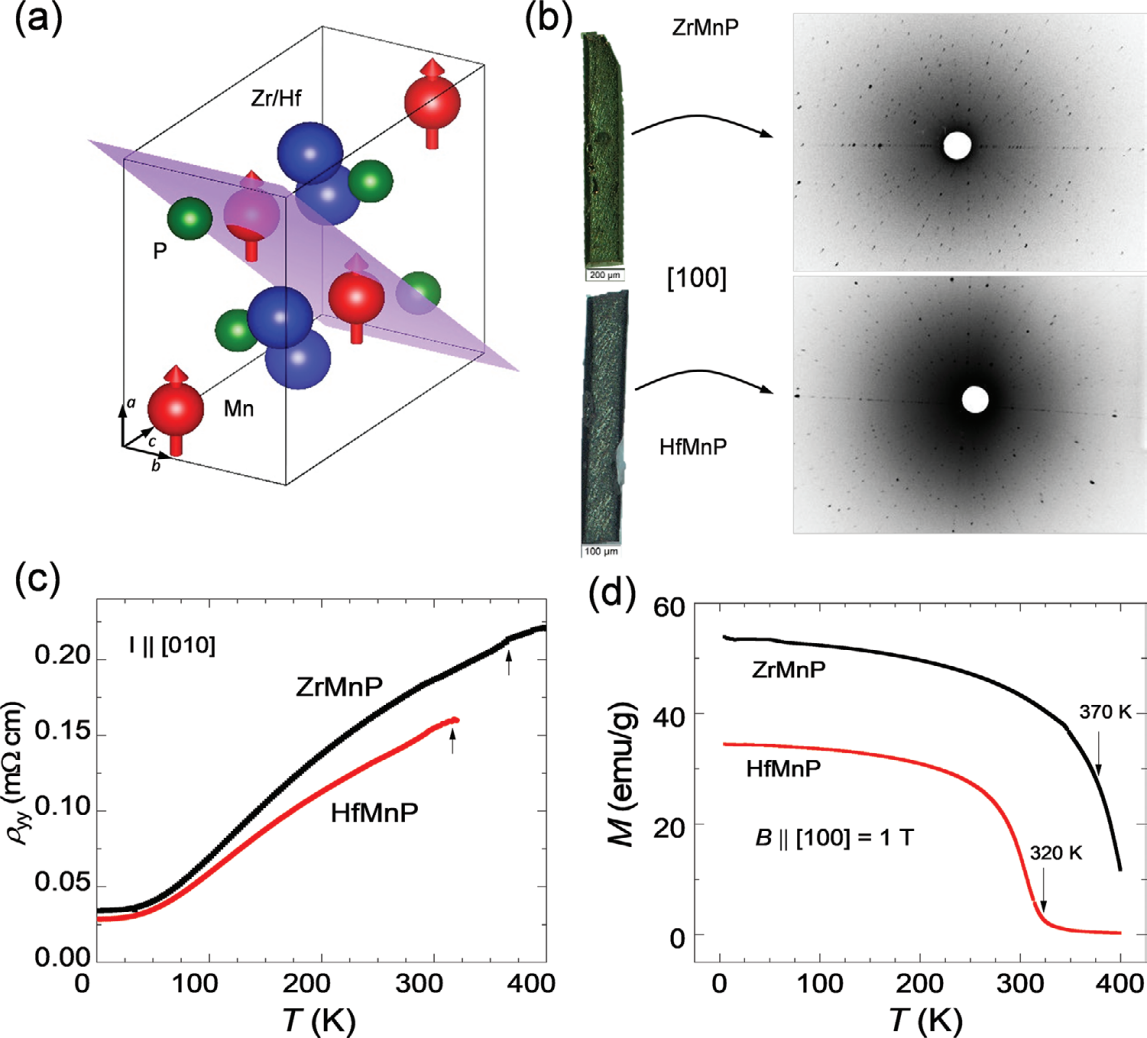
Crystal structure, Laue pattern, resistivity, and magnetic measurements of ZrMnP and HfMnP. a) Orthorhombic unit cell of compounds with SG *Pnma*. The highlighted (101) is one of the mirror planes. b) [100] facet of as‐grown crystals and their corresponding Laue patterns, where the needle direction is [010]. c) Temperature‐dependent longitudinal resistivity ρ_*yy*_ along [010], indicating a metallic character with the kinks (indicated by arrows) corresponding to the ferromagnetic transition temperature (*T*_C_) of the compounds. d) Temperature‐dependent magnetization along [100] with *T*_C_ indicated by arrows.

Transition metal pnictides are of significant interest as they possess both a high ferromagnetic transition temperature (*T*_C_) above room temperature and large magnetic anisotropy. ZrMnP and HfMnP are particularly important compounds, which crystallize in a TiNiSi‐type orthorhombic structure with space group (SG) *Pnma* (No. 62). Single‐crystal X‐ray analyses demonstrated the lattice parameters to be *a* = 3.64 Å, *b* = 6.45 Å, and *c* = 7.53 Å for ZrMnP and *a* = 3.61 Å, *b* = 6.38 Å, and *c* = 7.47 Å for HfMnP. Both ZrMnP and HfMnP are ferromagnets and their observed *T*_C_ values are 320 and 370 K, respectively (**Figure** **1**d), which match well with the previous report.^[^ ^17^ ^]^ Only the Mn atoms contribute to the magnetism (as indicated in Figure 1a). The crystals of ZrMnP and HfMnP grew in a needle shape along the *b*‐axis with well‐defined facets, as demonstrated by the Laue X‐ray diffraction (Figure 1b). However, the *a*‐ and *c*‐axes were the easy and difficult directions for the magnetic moments, respectively, resulting in large magnetic anisotropy.^[^ ^17^ ^]^ The measured values of the saturation magnetization at 2 K were 1.8μ_B_ f.u.^−1^ for ZrMnP and 2.0μ_B_ f.u.^−1^ for HfMnP, which is consistent with previously reported value.^[^ ^17^ ^]^ Our first‐principles calculations revealed that these compounds possess Dirac nodal lines, which are protected by the various mirror planes at different energies.

## 2 Results and Discussion

The zero‐field longitudinal resistivity ρ_*yy*_ (Figure 1c) of both compounds increased as the temperature increased, indicating metallic behavior. For the current *I*∥[010], the value of ρ_*yy*_ at 2 K was 3.4 × 10^−5^ Ω cm for ZrMnP and 2.9 × 10^−5^ Ω cm for HfMnP. Both compounds exhibited similar values of the residual resistivity ratio [*RRR* = ρ_*yy*_ (300 K)/ρ_*yy*_(2 K)], which was 5.5, reflecting high quality of the crystals. The kinks in their respective resistivity measurements corresponded to the magnetic transition.^[^ ^17^ ^]^ A magnetic transition from the ferromagnetic to paramagnetic state was observed in the temperature‐dependent magnetic measurements with a magnetic field *B* = 1 T along [100], as illustrated in Figure 1d. ZrMnP exhibited a relatively higher *T*_C_ (370 K) than that of HfMnP (320 K), which was consistent with the resistivity measurements. Throughout this manuscript, the *a*‐, *b*‐, and *c*‐axes of the compounds are equivalent to the *x*‐, *y*‐, and *z*‐directions in the measurements, respectively.

**Figure 2:**
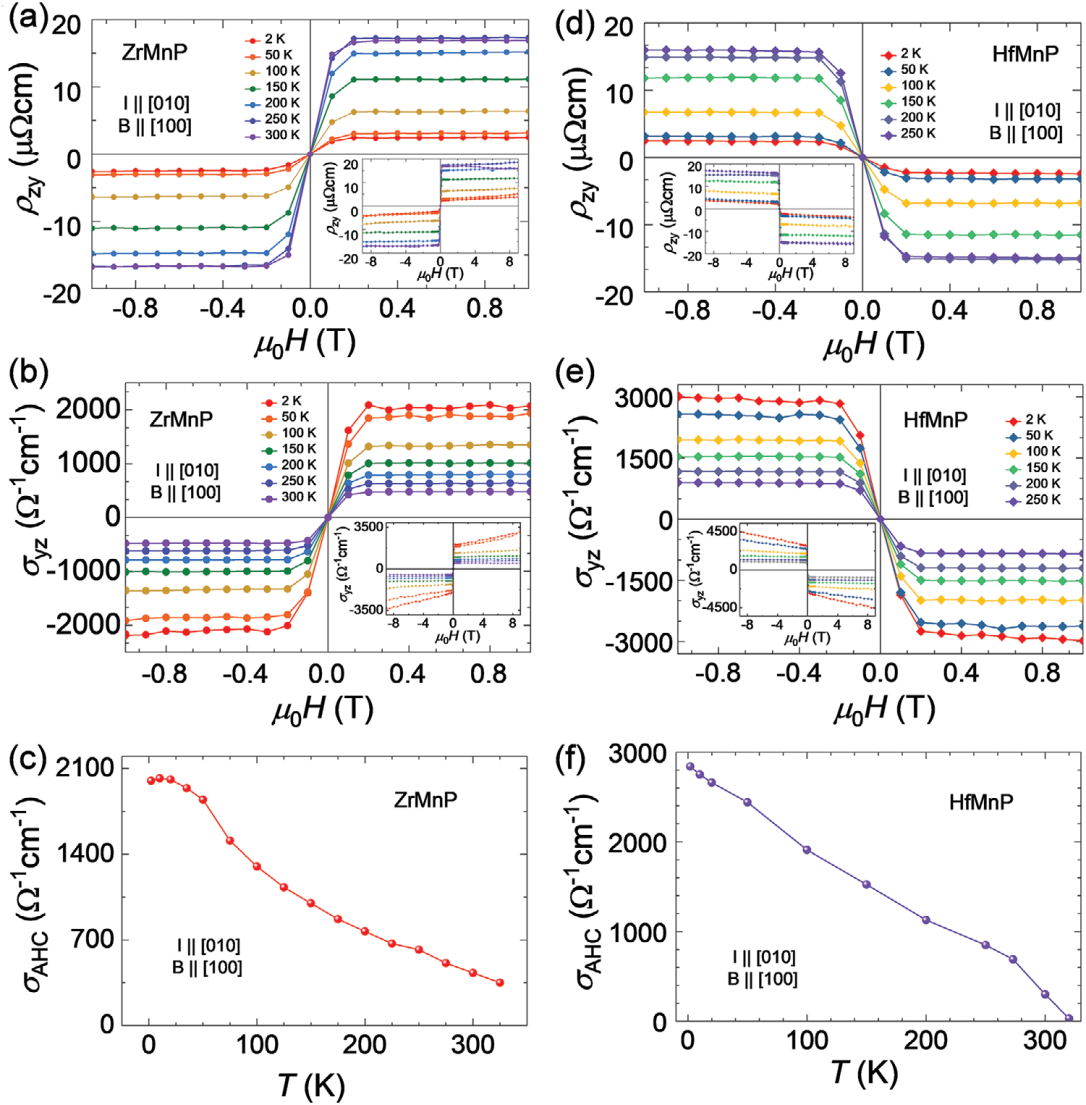
Hall resistivity ρ_*zy*_ and conductivity σ_*yz*_ of ZrMnP and HfMnP when field **B**∥**a** and current **I**∥**b** in the field range of ±1 T. The left‐column panels for ZrMnP (a–c): a) field‐dependent measured ρ_*zy*_ at several temperatures and b) corresponding calculated σ_*yz*_ from relation $\sigma_{yz}=\frac{\rho_{zy}}{\rho_{zy}^{2}+\rho_{yy}^{2}}$, and c) temperature‐dependent extracted σ_AHC_. The same measurements are provided in the right‐column panels (d–f) for HfMnP. The corresponding insets are the data in the field range of ± 9 T.

**Figure 3:**
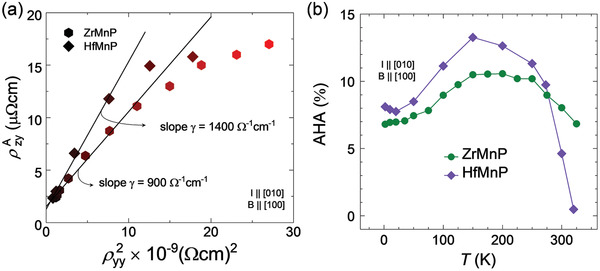
Unified scaling law to extract intrinsic AHC and anomalous Hall angle (AHA). a) Plot of AHE versus $\rho_{yy}^{2}$ at various temperatures, where the linear slope indicates the intrinsic AHC value. Refer to the text for the deviation from the linearity at the higher end. b) Temperature‐dependent measured AHA for ZrMnP and HfMnP, where AHA is defined as the ratio σ_AHC_/σ_*yy*_.

As illustrated in Figure 1, the grown crystals of both compounds had a needle shape, which provided the opportunity to perform Hall measurements without any additional work of cutting the crystals in a Hall bar geometry. The magnetic field‐dependent Hall resistivity ρ_*zy*_ was measured at various temperatures in the range of −9 to 9 T. The observed behavior of ρ_*zy*_ is presented in **Figure** **2**a,b, where the current was passed along the *b*‐axis and the magnetic field was applied along the *a*‐axis; that is, the easy axis of magnetization. The sign of the measured Hall resistivity data for the ZrMnP indicates that holes constituted the majority of the charge carriers, whereas in the case of HfMnP, the majority of charge carriers were electrons. These findings are in good agreement with the numerical results from the density functional theory (DFT) calculations (see band structures below, later in Section 2). A hole pocket exists around the *U*‐point in the first Brillouin zone, which cuts the Fermi energy (*E*_F_) for ZrMnP but not for HfMnP. An anomaly is observed in the ρ_*zy*_ measurements, which is attributed to the anomalous Hall effect (AHE) that normally appears in metallic ferromagnets; that is, a sharp increase at a lower field, followed by saturation with a further increase in the field. Moreover, it exhibits a resemblance with the magnetization curve; however, its saturation value decreases with a decrease in the temperature. The measured ρ_*xy*_ in ferromagnets is usually defined as a combination of two terms: ρ_*xy*_(*T*) = ρ_OHE_(*T*) + ρ_AHE_(*T*), where ρ_OHE_ is the contribution from the ordinary Hall effect that arises from the Lorentz force acting on the charge carriers, whereas ρ_AHE_ is the anomalous Hall contribution, which is unique to magnetic samples.^[^ ^18^ ^]^ The value of ρ_AHE_ at 2 K is 2.5 μΩ cm for ZrMnP and 2.3 μΩ cm for HfMnP (Figure 2a,d), which indicates an increasing trend, and reaches 17.2 μΩ cm and 16 μΩ cm, respectively, at 250 K. The ρ_AHE_ value at a fixed temperature was estimated by interpolating the high field value of ρ_*zy*_ to the zero‐field as the y‐intercept. Similarly, the AHC σ_AHC_ was estimated from the Hall conductivity $\left(\sigma_{yz}=\frac{\rho_{zy}}{\rho_{zy}^{2}+\rho_{yy}^{2}}\right)$ as the *y*‐intercept. The σ_AHC_ at 2 K is 2000 Ω^−1^ cm^−1^ for ZrMnP and 2840 Ω^−1^ cm^−1^ for HfMnP (Figure 2b,e), which is observable up to their transition temperature (Figure 2c,f). The total σ_AHC_ has both intrinsic and extrinsic contributions from different mechanisms. The intrinsic contribution originates from the electronic band structure (the BC), whereas the extrinsic contribution arises from the skew scattering or side‐jump effect. To define these contributions empirically, the temperature‐dependent estimated $\rho_{xy}^{A}$ can be expressed as $\rho_{xy}^{A}(T)=\alpha \rho_{xx0}+\beta \rho_{xx0}^{2}+\gamma \rho_{xx}^{2}(T)$, where ρ_*xx*0_ is the residual longitudinal resistivity.^[^ ^18^ ^]^ The first and second terms represent the extrinsic contributions from the skew scattering and side‐jump, respectively, whereas the final term denotes the intrinsic contribution from the BC that is associated with nontrivial bands. To extract the intrinsic contribution, we plotted $\rho_{zy}^{A}$ as a function of $\rho_{yy}^{2}$ at different temperatures. The slope γ of the straight line directly provides the estimation of the intrinsic value of AHC, which is ≈900 Ω^−1^ cm^−1^ for ZrMnP and 1400 Ω^−1^ cm^−1^ for HfMnP (**Figure** **3**a). It can be observed that the $\rho_{zy}^{A}$ versus $\rho_{yy}^{2}$ plot down‐turns slightly with an increasing temperature. The origin of such deviation is not very clear and it has also been observed in other compounds.^[^ ^8^, ^12^, ^15^, ^19^, ^20^ ^]^ However, the shifting of the chemical potential with the temperature is the most typical and persuasive explanation, whereby the AHC is constant only through the SOC gap.^[^ ^21^ ^]^

In compounds that possess a large BC‐induced AHC, a large AHA is also expected. The AHA defines the ratio of the AHC to the longitudinal conductivity σ_*yy*_ at the zero‐field: AHA = σ_AHC_/σ_*yy*_ (*B* = 0). The AHA exhibits a linearly increasing trend with an increase in the temperature, but decreases further after peaking at ≈150 K. This is because as *T*_C_ is approached, the anomalous behavior begins to diminish as the material starts to lose its ferromagnetic property. Both ZrMnP and HfMnP exhibit very high AHA values of 10.2% and 13.6%, respectively (Figures 3a,b). These values are sufficiently high to maintain the present compounds among the various ferromagnetic nodal line compounds.^[^ ^8^, ^12^, ^15^, ^19^ ^]^ Notably, ZrMnP and HfMnP had the highest AHC values to the best of our knowledge as well as relatively high AHA values.

**Figure 4:**
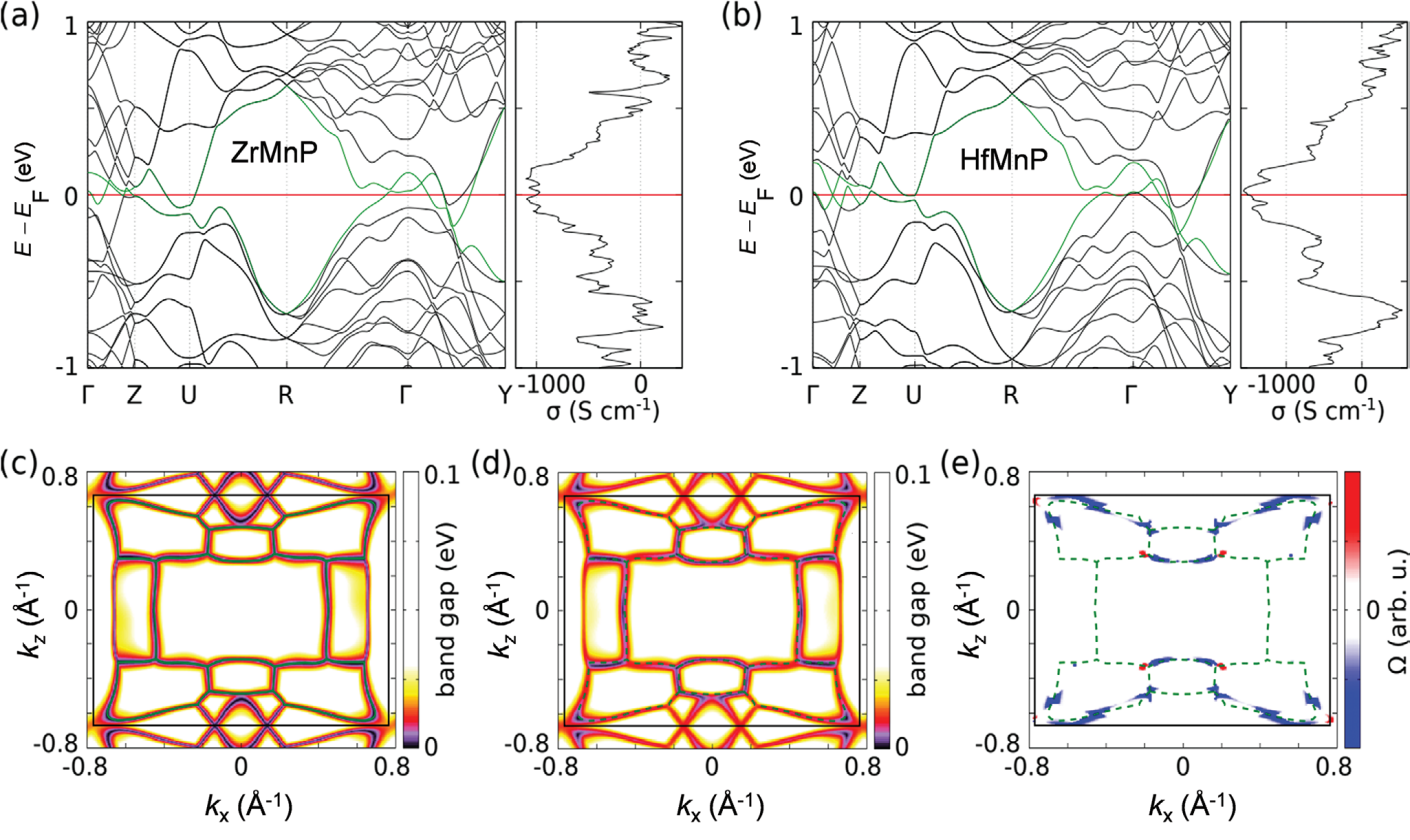
Band structure, AHC, and BC of ZrMnP and HfMnP. a) Band structure and AHC of ZrMnP. b) Band structure and AHC of HfMnP. In both (a) and (b), the bands (green lines) which contribute to forming nodal‐lines c) Bandgap of bands marked in green of HfMnP in (b) in *y* = 0 plane without SOC. Several closed loops are visible (green lines). d) *y* = 0 plane with SOC and magnetization along the *a*‐axis, with gapped nodal lines (dashed green lines). e) Berry curvature Ω, at *E*_F_ in the *y* = 0 plane. The large contributions are located around the gapped nodal lines (dashed green lines). Similar results to those in (c–e) are also expected for ZrMnP.

After measuring the AHC values, we applied the tight‐binding method (see the Experimental Section for details) to understand the origin of the AHC. The calculated band structures for the ZrMnP and HfMnP are presented in **Figure** **4**a,b, respectively. Except a tiny band along Γ–*Y* (black line), only those bands (green lines) appear at the *E*_F_ which contribute to the nodal‐lines. Such bands primarily dominate in electrical transport. There are three mutually perpendicular mirror planes for the particular SG symmetry *Pnma*. Our calculations without SOC reveal several nodal loops that are located in the band structure, which are enforced by these mirror planes (Figure 4c). By considering the SOC and magnetization direction along the *a*‐axis as in the experiments, the magnetic moments are not compatible with the mirror symmetry at *y* = 0.^[^ ^8^, ^9^ ^]^ In this scenario, the nodal lines are no longer protected and the degeneracy is lifted (Figure 4d). This, in turn, creates strong BC contributions along the former nodal lines, as illustrated in Figure 4e, which directly contributes to the intrinsic part of the AHC. The calculated intrinsic parts of the AHC are ≈1000 Ω^−1^ cm^−1^ for ZrMnP and ≈1500 Ω^−1^ cm^−1^ for HfMnP, which are in excellent agreement with the intrinsic parts of the experimental values. As these values are remarkably high, the studied compounds are interesting cases among the known nodal‐line compounds.

Among the AHC values that were measured in different directions, the highest values were observed when *B*∥*a*. In this scenario, all nodal lines corresponding to the mirror plane at *y* = 0 has their degeneracy lifted and the nontrivial gap is opened, whereas the other nodal lines remain degenerate. As this gap is larger for HfMnP, we observed a larger AHC for HfMnP than for ZrMnP. Our previous study indicated that the number of mirror planes present in the compound plays a crucial role in the manipulation of the AHC.^[^ ^10^ ^]^ This was demonstrated by using the simple example of two different space groups that possessed different numbers of mirror planes. SG 225 contains three mutually perpendicular mirror planes at *x* = 0, *y* = 0, and *z* = 0, whereas these planes are absent in SG 216. Consequently, the compounds that are associated with SG 225 exhibit larger AHC values compared to SG 216. Moreover, the location of the nodal line is an important factor. Naturally, the nodal line structure can only contribute to the transport if it is located at *E*_F_. In these two cases, the number of mirror planes is related to the crystal structure, whereas the location is material specific. Our present investigation suggests that compound selection aimed at a high intrinsic value of AHC can be achieved by the tuning of the mirror symmetry that is inevitably present in various achiral space groups.

## 3 Conclusion

We have demonstrated AHE in ferromagnetic ZrMnP and HfMnP. The crystal structure of these compounds comprises numerous mirror planes, which result in gapped nodal‐line states in the band structure under the SOC effect. Among the various nodal lines, several lie at the Fermi energy, making these compounds unique compared to other known nodal‐line compounds. These nodal lines are directly responsible for high AHC values owing to the accumulation of large BCs. Various advantages are offered by the present selection of compounds, such as a large ferromagnetic transition temperature, one of the largest ever intrinsic AHC values, a significantly large AHA, and the scope to observe the SOC effect on the AHC value. Our investigations provide various opportunities for tuning the AHC as well as searching for more compounds with higher numbers of mirror planes in their crystal structures.

## 4 Experimental Section

### Crystal Growth and Characterization

The single crystals of ZrMnP and HfMnP were grown by the self‐flux method, similar to that reported by Lamichhane et al.^[^ ^17^ ^]^ Initially, Mn chips (99.99%) were cleaned by placement in an evacuated quartz tube, heated to 1000 °C at a rate of 200 °C h^−1^, and maintained for 24 h before switching off the furnace. Shiny silver Mn pieces were obtained, which were subsequently ground into powder. For the single crystal growth, grounded Mn powder, P lumps (99.999%), and small pieces of Zr/Hf from their foils (99.8%) were used in a (Zr/Hf)_1.25_Mn_85.9_P_12.85_ stoichiometry ratio and placed in the dried alumina crucibles. Thereafter, the crucibles were sealed in a quartz tube with 5 mbar partial pressure of argon. The entire reaction mixture was placed in a box‐furnace and heated in two steps: first to 250 °C at a rate of 50 °C h^−1^ and maintained for 3 h, and subsequently to 1180 °C at the same heating rate. At 1180 °C, the reaction was maintained for 12 h for homogeneity. Slow cooling to 1025 °C was conducted over 180 h, at which time the additional flux was removed with centrifugation. Silver needle‐shaped single crystals were obtained from the above reaction profile. The crystals of both the compounds were stable in air and moisture. Elemental analysis with energy dispersive x‐ray confirmed that the composition of the compounds was close to 1:1:1. The crystal orientation was checked in an X‐ray Laue diffractometer and the patterns were analyzed by using Orient Express software.

### Electric and Magnetic Measurments

The electric and magnetic measurements were performed in Quantum Design PPMS and MPMS instruments, respectively. The electrical resistivities were measured by standard four‐probe in ACT option. In order to get the zero Hall resistivity in zero field at a particular temperature, the offset value due contact misalignment was removed.

### Numerical Methods

For the theoretical investigations, ab initio calculations were employed based on DFT, as implemented in Vienna Ab initio Simulation Package (VASP).^[^ ^22^ ^]^ This code uses plane waves and pseudopotentials as a basis set. The exchange‐correlation potential was used in the generalized gradient approximation.^[^ ^23^ ^]^ The *k* mesh used for the integration over the Brillouin zone was 7 × 13 × 7. For calculations with a denser *k* mesh, Wannier functions were extracted from the DFT results using the Wannier90 package.^[^ ^24^ ^]^ Using these Wannier functions, a tight‐binding Hamiltonian H was constructed, which was used to evaluate the Berry curvature Ω in the system as follows^[^ ^18^, ^21^, ^25^ ^]^

(1) $$\Omega_{xy}^{z}(n)=i\underset{m\neq n}{\sum }\frac{\left\langle n\left|\frac{\partial H}{\partial k_{x}}\right|m\right\rangle \left\langle m\left|\frac{\partial H}{\partial k_{y}}\right|n\right\rangle ‐x↔y}{{\left(E_{n}-E_{m}\right)}^{2}}$$

where |*n*〉 and *E_n_* are the eigenstates and energies of Hamiltonian *H*. On this basis, the AHC was calculated as follows^[^ ^18^, ^21^ ^]^

(2) $$\sigma_{xy}=‐\frac{e^{2}}{\hbar }\underset{n}{\sum }\int \frac{d^{3}k}{{\left(2\pi \right)}^{3}}f_{n}\Omega_{xy}^{z}(n)$$

where *f_n_* is the Fermi distribution function. The *k* mesh for the integration over the Brillouin zone in this step was selected as 301 × 301 × 301 to ensure converged results.

## Conflict of Interest

The authors declare no conflict of interest.

## Data Availability

The data that support the findings of this study are available from the corresponding author upon reasonable request.
